# Perspectives: Mental health challenges and medical trauma: focus on cystinosis patients and caregivers

**DOI:** 10.3389/fped.2025.1592513

**Published:** 2025-05-29

**Authors:** Maya H. Doyle, Chelsea Meschke

**Affiliations:** ^1^ Department of Social Work, School of Health Sciences, Quinnipiac University, Hamden, CT, United States; ^2^ Family Support, Cystinosis Research Network, Lake Forest, IL, United States

**Keywords:** cystinosis, rare disease, mental health, pediatric medical trauma, caregiving, medical trauma

## Abstract

As the lifespan of cystinosis patients extends, mental health issues increasingly impact both patients and their caregivers. The emotional journey for patients and “raregivers” has been mapped, providing a valuable guide across a patient's lifetime. Common mental health challenges within the cystinosis community include anxiety, depression, PTSD, grief/loss, bullying, peer challenges, body image issues, and underappreciation of gender and sexual identity, alongside co-occurring mental health diagnoses. A literature review was conducted and a first presentation given on this under-appreciated topic at the New York Academy of Medicine in May 2024. Pediatric Medical Trauma is documented in the literature; however, post-traumatic stress disorder (PTSD) related to medical trauma and caregiving across the lifespan for both patients and carers is often subsumed under general stress, leading to a lack of targeted interventions. Medical events, from ICU experiences to routine blood draws, can be traumatic or trigger past trauma, resulting in classic PTSD symptoms. Despite referrals for mental health services, there is often a lack of awareness about specific interventions for post-traumatic stress, such as TF-CBT, DBT, EMDR, ACT, and other forms of mindfulness-based stress reduction. Healthcare teams are encouraged to monitor for signs of mental health distress, including reduced adherence and changes in appearance, affect, and demeanor towards care. Proactive conversations and anticipatory guidance are essential to educate and normalize mental health care needs. Ensuring patients and families are aware of available resources through disease-specific organizations, hospital services, and local mental health agencies is vital.

## Introduction

Pediatric Medical Trauma (PMTS) is well-documented in the literature for children with many chronic conditions (1–3). Medical events, from ICU experiences to routine blood draws or the g-tube changes described in our case study, can be traumatic or trigger past trauma. Chronic and daily experiences related to illness can also be perceived as traumatic, even outside of the medical setting (1), and may include parts of the care regimen itself. Psychological stressors related to accessing care and navigating the healthcare system, insurance struggles around authorizations or coverage, or financial toxicity (4) related to the high economic impact of rare condition (5) can also create feelings of anger, fear, and overwhelm. Classic PTSD symptoms, including re-experiencing and flashbacks, nightmares, avoidance, and hypervigilance/hyperarousal may go unrecognized, or simply accepted as part of the “new normal” for patients and families. These symptoms may increase fear or avoidance of care (or care locations) or non-adherence (6) with recommended regimen.

## Trauma-informed medical care

The concepts of trauma-informed care have expanded from mental health and crisis settings to use in schools, shelters, corrections, specific medical settings such as HIV care and maternity, and other programs serving youth and adults. Understanding has grown of how trauma impacts how individuals may approach, access, or utilize services (7). The psychological and physiological sequelae of trauma, whatever its root cause, and at times unrecognized by the individual, may be better recognized clinically. Recommendations for using a trauma-informed approach to medical care (3, 8) include things that healthcare teams already do (or aspire to do) such as patient-centered care and communication, and interprofessional collaboration, combined with an increased knowledge of the impact of trauma on health, greater awareness and self-reflection regarding one's own history and responses to trauma and crisis, and improved screening mechanisms for medical trauma. Techniques and resources for primary care and specialty settings and for healthcare providers themselves are proliferating (9).

## Pre-emptive strategies to minimize trauma

During hospital stays or pediatric subspecialty visits, involvement of social work to regularly monitor patient and family well-being can support other members of the team in this crucial element of care. Both social work and child life specialists (10) can engage pediatric patients, as well as siblings (11), in supportive interventions and therapeutic play (12), to support understanding and developmentally-appropriate coping and self-expression. Numerous members of the health care team, including physicians, nurses, occupational and physical therapy, and pharmacy can offer education and anticipatory guidance to make care events and routines more tolerable.

## Appropriate referrals

Post-traumatic stress disorder (PTSD) related to medical trauma across the lifespan for both patients and carers is often subsumed under general stress, leading to a lack of recognitions or targeted interventions (13). Despite referrals for mental health services, there is often a lack of awareness about specific interventions for post-traumatic stress. Non-adherence, one of the most challenging problems for a healthcare team and family, when viewed through a trauma lens, might be considered (for some) a form of self-harm again to cutting or suicidal thoughts. There are numerous third-wave cognitive-behavioral interventions which balance change and acceptance are appropriate for addressing the symptoms and behaviors related to medical trauma:

• Trauma-focused cognitive-behavioral therapy TF-CBT) (14, 15)—an intervention developed for children and adolescents impacted by developmental traumas, as well as caregivers, to address distorted/distressing beliefs and attributions, and acquire more adaptive coping skills.
• Dialectic Behavior Therapy (DBT) (16)—a structured individual or group intervention with a focus on distress tolerance, emotional regulation, interpersonal effectiveness, and mindfulness, particularly oriented toward those with self-harming behaviors, suicidal ideation, and high-risk non-adherence.
• EMDR (17)—an intervention utilizing guided eye movements or other bilateral stimulation such as tapping, pulsing, or sound while processing traumatic memories.
• Acceptance & Commitment Therapy (ACT) (18)—an intervention to promote psychological flexibility through the acceptance of thoughts, emotions, and sensations (including pain) while cultivating awareness of the present and a stable sense of self, and one's values, even in the face of difficult circumstances, including chronic and life-altering illness.
• mindfulness-based stress reduction (19, 20)—a meditative and attention-shifting practice developed specifically to help people manage pain and stress associated with long-term conditions.
• polyvagal-oriented therapies (21)—interventions which helps increase awareness of the nervous system response to stress and danger, and empowers individuals to use meditative and somatic strategies to manage such response and process trauma.

## The example of cystinosis

Cystinosis is a rare metabolic disease (prevalence), markedly impacted growth and kidney function As the lifespan of cystinosis patients extends (22–24), mental health issues increasingly impact both patient and caregiver well-being and quality of life across the lifespan. While many aspects of living with cystinosis may become normalized by patients and families over time (25), specific incidents of crisis and ongoing stressors may be considered traumatic and overwhelm the coping mechanisms of the patient or family.

In preparing for a symposium on cystinosis held at the New York Academy of Medicine in May 2024 with a multidisciplinary audience of healthcare providers, patients and family members, a brief literature review was conducted and a presentation on this under-appreciated topic developed by the authors (an experienced pediatric nephrology social worker and a cystinosis parent) at the New York Academy of Medicine in May 2024. The following case ezampleis adapted from a cystinosis family to illustrate the impact of illness and diagnosis for one child and family.

The emotional journey for patients and “raregivers” has been mapped, providing a valuable guide across a patient's lifetime (26). Parents enduring the lengthy or frightening “diagnostic odyssey” or life-threatening events for their child common to rare disorders may experience post-traumatic symptoms of hypervigilance, dissociation, and flashbacks (27, 28). Mental health challenges discussed within the cystinosis community, by pediatric and adult patients and by families and partners, include anxiety, depression, reduced adherence with medical regimen, grief/loss, bullying, peer challenges, body image issues, underappreciation of gender-identity and sexual orientation, alongside co-occurring mental health diagnoses (29).

## Case study

James is a 7-year-old boy diagnosed with cystinosis at 6 months old. His pediatrician identified Renal Fanconi Syndrome through urinalysis and referred the patient and family to subspeciality care with nephrology and genetics teams for full work-up and diagnosis, who confirmed James's diagnosis of nephropathic cystinosis. Medications were started immediately with follow-up every two to three months to ensure levels were improving and stabilizing. After four months, the healthcare team encouraged the placement of a gastronomy tube (g-tube) to assist with additional supplement intake as well as medication adherence (a common intervention for children with cystinosis). To the family, the explanation and discussion around this plan, though perhaps routine to the team, felt uncaring and “simple”. This early in their rare disease journal, even the simplest procedures weighed as large decisions for which they felt unprepared.

Due to scheduling delays, a nasogastric (NG) tube was placed for 2–3 weeks before the surgery. Having to provide such care at home for a 10-month-old was overwhelming to both parents, but they undertook what was necessary for their child's well-being. Once the g-tube was placed, it was changed routinely every 3 months at the hospital, an hour's ride from home, for the first 9 months, and then family was perfunctorily trained to change it themselves at home. Positively in that time, James's growth and weight gain improved, and he could more easily tolerate feedings and multiple medications.

Though necessary for his well-being, this repeated g-tube change at home became highly stressful for both James and his parents. The moment he saw the preparations and supplies for the g-tube change, he would hide, then scream and cry, eventually requiring one parent to hold his limbs while the other changed the tube (in about 30 s). When James's younger brother Max was born, he was also diagnosed with cystinosis quickly after birth. His parents agreed to a g-tube placement at 4 months old for Max, as they debated the potential challenge of “forcing” medications and oral feedings (which they had seen impact James's relationship with food) vs. g-tube use and changes. The boys, now 3 and 7, are doing well overall, growing, enjoying eating, and with adequate management of their cystinosis. However, both continue to require a g-tube change every 3 months

Their mother describes her own anxiety before, and exhaustion, sadness, and guilt afterward for causing her children distress. Though the g-tube changes are more normalized now, they remain challenging; she wonders how these interactions will impact them later and if it makes home and she herself feel less safe to her sons. She self-referred to mental health providers for her own talk therapy regarding the burden, sadness, and shame she was experiencing, along with anti-anxiety medications and reaching out for peer support from other cystinosis and rare parents.

Over time, the family has created or identified helpful strategies to make these difficult episodes more tolerable, many of which echo elements of the interventions described above. Regretfully, these suggestions were not forthcoming from their healthcare team.

• Simple straightforward, timely communication (about a hour's lead time before g-tube change)
• Letting the older child see the removed g-tube and how “yucky” it is.
• breathing techniques and calming exercises
• token reward system (or as mother reports, “bribery”)
• acknowledgement that the g-tube change is uncomfortable and difficult
• Audio or video distraction
• A reassuring “selfie” video made by older child about what the experience is like
• An “emotional toolbox” of fidget toys and comfort items

## Conclusion

Healthcare professionals providing care to pediatric cystinosis patients and other rare and/or chronic illnesses must increasingly be aware of the mental health impact of illness (and treatment) upon both pediatric patients as they mature, as well as their entire family. While that impact may manifest immediately as a result of a traumatic diagnostic experience or medical crisis, it can also reverberate across the lifespan and impact coping and decision-making for patients themselves and caregivers for years. As healthcare professionals, it is necessary to consider how we can provide trauma-informed medical care, engage in pre-emptive strategies to minimize trauma, assess for and recognize symptoms of post-traumatic stress, and destigmatize the mental health challenges that arise amidst chronic and rare health conditions like cystinosis.

This article seeks to increase awareness of the topic of medical trauma using the cystinosis experience as an example, and to offer practical suggestions for pre-emptive and post-traumatic intervention. Healthcare teams are encouraged to monitor for signs of mental health distress, including reduced adherence and changes in appearance, affect, and demeanor towards care. Proactive conversations and anticipatory guidance are essential to educate and normalize mental health care needs. Ensuring patients and families are aware of available resources through disease-specific organizations, hospital services, and local mental health agencies is vital. Suggestions for future research would include disease and treatment-modality specific screening tools for medical trauma, consideration of the impact of PMTS on the transition to adulthood and adult care, and the longitudinal impact of trauma on young people with special healthcare needs and their family members and ongoing evaluation of trauma-informed care and interventions.

## Data Availability

The original contributions presented in the study are included in the article/Supplementary Material, further inquiries can be directed to the corresponding author.
